# An integrated approach to diagnosis and management of severe haemoptysis in patients admitted to the intensive care unit: a case series from a referral centre

**DOI:** 10.1186/1465-9921-8-11

**Published:** 2007-02-15

**Authors:** Muriel Fartoukh, Antoine Khalil, Laurence Louis, Marie-France Carette, Bernard Bazelly, Jacques Cadranel, Charles Mayaud, Antoine Parrot

**Affiliations:** 1 Service de Pneumologie et Unité de Réanimation Respiratoire, Hôpital Tenon, Assistance Publique – Hôpitaux de Paris and Université Pierre et Marie Curie, 4 Rue de la Chine, 75020 Paris, France; 2 Service de Radiologie, Hôpital Tenon, Assistance Publique – Hôpitaux de Paris and Université Pierre et Marie Curie, 4 Rue de la Chine, 75020 Paris, France; 3 Service de Chirurgie Thoracique et Vasculaire, Hôpital Tenon, Assistance Publique – Hôpitaux de Paris and Université Pierre et Marie Curie, 4 Rue de la Chine, 75020 Paris, France

## Abstract

**Background:**

Limited data are available concerning patients admitted to the intensive care unit (ICU) for severe haemoptysis. We reviewed a large series of patients managed in a uniform way to describe the clinical spectrum and outcome of haemoptysis in this setting, and better define the indications for bronchial artery embolisation (BAE).

**Methods:**

A retrospective chart review of 196 patients referred for severe haemoptysis to a respiratory intermediate care ward and ICU between January 1999 and December 2001. A follow-up by telephone interview or a visit.

**Results:**

Patients (148 males) were aged 51 (± sd, 16) years, with a median cumulated amount of bleeding averaging 200 ml on admission. Bronchiectasis, lung cancer, tuberculosis and mycetoma were the main underlying causes. In 21 patients (11%), no cause was identified. A first-line bronchial arteriography was attempted in 147 patients (75%), whereas 46 (23%) received conservative treatment. Patients who underwent BAE had a higher respiratory rate, greater amount of bleeding, persistent bloody sputum and/or evidence of active bleeding on fiberoptic bronchoscopy. When completed (n = 131/147), BAE controlled haemoptysis in 80% of patients, both in the short and long (> 30 days) terms. Surgery was mostly performed when bronchial arteriography had failed and/or bleeding recurred early after completed BAE. Bleeding was controlled by conservative measures alone in 44 patients. The ICU mortality rate was low (4%).

**Conclusion:**

Patients with evidence of more severe or persistent haemoptysis were more likely to receive BAE rather than conservative management. The procedure was effective and safe in most patients with severe haemoptysis, and surgery was mostly reserved to failure of arteriography and/or early recurrences after BAE.

## Background

Haemoptysis may present as a life-threatening condition, with a mortality rate reaching 80% in the absence of adequate and prompt management [1-4]. The criteria used to characterize severe haemoptysis are heterogeneous and ill-defined. They are usually limited to the amount of blood expectorated within 24–48 hrs and its clinical consequences [5], or to the interventions used [6]. A more 'functional' definition accounting for the respiratory reserve has also been proposed [6]. Recent surveys suggest a shift from surgery to bronchial artery embolisation (BAE) as a first-line procedure in severe haemoptysis [7,8]. Defining a better standardized management would be useful to physicians in charge of patients with severe haemoptysis to improve outcomes and should preferably take place in or nearby the intensive care unit (ICU).

In this study, we analyzed a large series of unselected patients with severe haemoptysis referred to a single respiratory intensive care unit with an affiliated intermediate care ward. Our objectives were to describe the characteristics of the patients managed using one of the three main initial therapeutic options (conservative measures, BAE or surgery) and to help better define the role of BAE, according to the severity of haemoptysis. The study was conducted in accordance with French law, which does not require approval of an IRB or the consent of patients for such retrospective analysis of medical records.

## Patients and methods

### Patients

The study was conducted between January 1999 and December 2001 in Tenon hospital, a tertiary university hospital and referral centre for haemoptysis in Paris, France. All consecutive patients admitted to the respiratory intermediate care ward or ICU for severe haemoptysis were eligible. Exclusion criteria were iatrogenic bleeding, bleeding of gastrointestinal and oropharyngeal origin, heart failure, intra alveolar haemorrhage and incomplete data. For each patient, the following information were recorded: baseline demographics, comorbid conditions, initial clinical presentation and vital signs, laboratory tests results, chest radiography, fiberoptic bronchoscopy and CT scan findings when performed, severity of haemoptysis, and pre-ICU and in-ICU management. The persistence or recurrence of bleeding, the patients' ICU and hospital lengths of stay and their vital status at discharge were recorded, as well as the occurrence of long-term rebleeding. Patients with recurrent haemoptysis were included at the first episode only.

### Definitions

#### 1. Severity of haemoptysis

The severity of haemoptysis on admission was assessed according to (i) the cumulated amount of bleeding; (ii) the consequences of bleeding; (iii) and the presence of associated severe cardiovascular and pulmonary comorbidities. The cumulated amount of bleeding on admission was assessed from the onset of bleeding until the first hours of admission to our unit using the following standardized scale: a spoonful (5 ml), a small filled glass (100 ml) and a large filled glass (200 ml). The consequences of bleeding were assessed on the need for administration of local or systemic terlipressin, mechanical ventilation, vasoactive drugs or blood transfusions before referral or within the first 24 hours of ICU admission.

#### 2. Cause of haemoptysis

The cause of haemoptysis was diagnosed on the combination of history, physical examination, chest radiography, fiberoptic bronchoscopy, CT scan, microbiology and histology when available. Definite causes were bronchiectasis (including inactive tuberculosis), active tuberculosis, cancer and mycetoma. Pulmonary venous thrombo-embolic disease, pneumonia and emphysema were classified as probable causes. Haemoptysis was considered cryptogenic when no cause was evidenced.

#### 3. Course of haemoptysis

Immediate control of bleeding was defined as a cessation of bleeding obtained without recurrence until hospital discharge, whatever the therapeutic option used. Rebleeding was defined as the persistence and/or the recurrence of bleeding after treatment. Early-onset rebleeding was defined as occurring within the first 30 days, and late-onset as rebleeding after one month.

### Management

Our approach to initial management favoured conservative measures and BAE over surgery, whenever possible. Conservative measures included strict bed rest, nothing by mouth, and continuous monitoring of oxygen saturation, respiratory rate, heart rate and arterial blood pressure. Oxygen was delivered to obtain a pulse oxymetry value > 90%; two large-bore intravenous lines were inserted and all medications potentially increasing the risk of bleeding were stopped. Broad-spectrum antibiotics were frequently administered and no attempt was made to suppress cough. Bronchoscopic techniques were attempted to control the bleeding, using cold saline solution lavage, instillation of topical vasoconstrictive agents and/or balloon tamponade therapy. As the administration of systemic terlipressin may interfere with the success of BAE, its use was avoided whenever possible.

The selection of BAE as the first-line approach was based on the presence of severity criteria on admission. A standardized BAE procedure was used as follows: a catheter was introduced into the right femoral artery through an introducer sheath using the Seldinger technique. A 5-French pigtail catheter (Angioflex, biosphere medical, Roissy, France) with the tip located at the origin of the ascending aorta was used, and 40 ml of contrast medium was administered at 20 ml/s. Selective bronchial artery angiography was then performed, using catheters ranging from 5 to 6.5 French. Embolisation was performed when the bronchial arteries appeared to be the source of haemoptysis (tortuous hypertrophy, systemic-to-pulmonary shunting, extravasation of contrast material, or peribronchial hyper-vascularisation) or when they had a near-normal aspect but supplied the site of bleeding identified by fiberoptic bronchoscopy and/or CT scan. The material used for embolisation was 400- to 1000-μm polyvinyl alcohol particles and/or gelfoam. A visualisation of an anterior spinal artery arising from an intercostal artery deriving from the right bronchointercostal trunk was considered an absolute contraindication to embolisation. Microcatheters were not used at the time of the study. BAE was considered successful when bleeding stopped immediately after embolisation.

### Statistical analysis

The patients' demographics, clinical variables and laboratory data were analyzed using usual descriptive statistics. Results were expressed as mean ± standard deviation (range), unless otherwise stated. Between groups comparisons used the Man Whitney *U *test for categorical variables, and the chi square test for nominal variables. A p value below 0.05 was considered statistically significant.

## Results

### Demographics, clinical features and biology

During the three-year study period, 230 consecutive patients were referred to our unit for severe haemoptysis. Thirty-four patients (15%) were excluded because of bleeding secondary to bronchial biopsies (n = 1), digestive tract bleeding (n = 1), pharyngeal bleeding (n = 1), heart failure (n = 3), intra alveolar haemorrhage (n = 2) and incomplete data (n = 26). Overall, 196 patients were thus included in this study. Most patients (n = 149, 76%) were referred to our unit from another hospital for consideration of BAE within 24 hours after hospital admission (1 ± 1.8 days; median 0) because haemoptysis persisted or worsened. The patients (148 males) were 51 years old. Cough, persistent bloody sputum and dyspnea were the main respiratory symptoms on admission. Physical examination revealed localized crackles in 60% of the cases (Table 1). There were mild biological consequences of bleeding regarding blood spillage and gas exchanges (Table 2).

**Table 1:** Clinical characteristics on ICU admission.

Age, years	51 ± 16 (17–89)
Sex Ratio (male:female)	148:48 (3.1:1)
SAPS II score	18 ± 9 (6–48)
McCabe and Jackson categories, n *	133/46/13
Cumulated volume of hemoptysis, ml †	240 ± 200 (10–1000)
< 200 ml, n (%)	86 (45%)
≥ 200 ml, n (%)	107 (55%)
Heart rate,/min	87 ± 20 (48–150)
> 130/min, n (%)	10 (5%)
Systolic Arterial Pressure, mm Hg	135 ± 28 (76–221)
< 100 mm Hg, n (%)	9 (5%)
Spontaneous Ventilation, n (%)	179 (91%)
Mechanical Ventilation [Invasive/Non Invasive], n (%)	17 [16/1] (9%)
Core Temperature, °C	37.3 ± 0.8 (36–40)
> 38.5°C, n (%)	18 (9%)
Respiratory functional signs	
Cough, n (%)	116 (73%)
Persistent bloody expectoration, n (%)	107 (69%)
Dyspnea, n (%)	121 (66%)
Purulent expectoration, n (%)	10 (6%)
Chest pain, n (%)	11 (6%)
Physical examination	
At least one localized abnormality, n (%)	94 (48%)
Crackles, n	59 (63%)

Results are expressed as mean ± SD (range), unless otherwise stated.

*4 missing data; †3 missing data.

**Table 2:** Biological variables on ICU admission.

Blood Leukocytes Count, mm^3^	9183 ± 3543 (1500–25300)
Platelets Count, mm^3^	258 464 ± 105 195 (45000–712000)
< 100 000/mm^3^, n (%)	6 (3%)
Hemoglobin, g/dl	12.6 ± 2.4 (4.6–18.3)
< 10 g/dl, n (%)	29 (15%)
Prothrombin Time, %	88 ± 16 (11–118)
≤ 50%, n (%)	7 (4%)
Activated partial thromboplastin time ratio	1.1 ± 0.2 (0.7–2.2)
≥ 1.5 control, n (%)	7 (4%)
Nitrogen Urea, mmoles/l	5.5 ± 2.7 (1–19)
≥ 10 mmoles/l, n (%)	12 (6%)
Blood gas on room air *	
PaO_2_, mm Hg	78 ± 17 (43–100)
PaCO_2_, mm Hg	39 ± 5 (26–63)
pH	7.43 ± 0.05 (7.30–7.50)
SaO_2_, %	95 ± 4 (76–99)

Results are expressed as mean ± SD (range), unless otherwise stated.

*data available for 142 patients.

### Severity of haemoptysis

**Figure 1:**
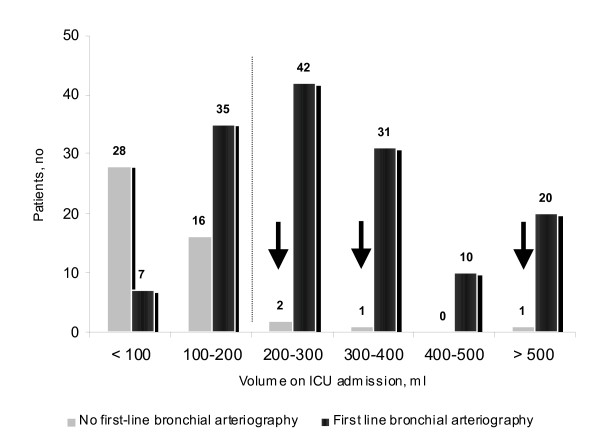
**Distribution of the cumulated volume of haemoptysis on ICU admission, according to the first attempt of bronchial arteriography**. Bronchial arteriography was not attempted in 4 patients with a volume ≥ 200 ml: one patient with moderate renal insufficiency (cryptogenic haemoptysis of 200 ml) received conservative treatment and emergency surgery was performed in the 3 other patients.

**Figure 2:**
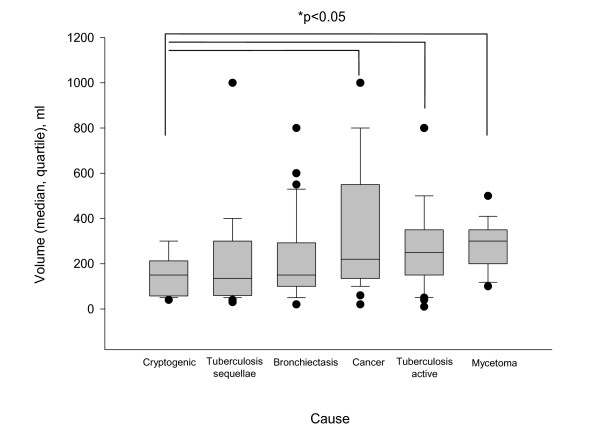
**Distribution of the volume of haemoptysis (median, quartile) on admission according to the cause**. Plots of the median, 10th, 25th, 75th, and 90th percentiles as vertical boxes with error bars.

Chronic obstructive pulmonary (n = 50, 26%) and/or cardiovascular (n = 53, 27%) disease were frequently recorded. Using our scale, the mean cumulated volume of blood loss averaged 240 ± 200 ml on admission to our unit (range, 10 to 1000 ml; median 200 ml) (Figure 1). Active tuberculosis, cancer and mycetoma were associated with a larger volume as compared with the cryptogenic group (p = 0.03; p = 0.03 and p = 0.003, respectively; Figure 2.). There were severe consequences of bleeding in 73 patients (37%) leading to the following interventions prior to the referral or during the first 24 hours of ICU admission: local (n = 23) or systemic (n = 56) terlipressin, mechanical ventilation (n = 17), blood transfusion (n = 22), vasoactive drugs support (n = 3) or cardiopulmonary resuscitation (n = 2). Patients receiving the above mentioned interventions had a higher respiratory rate on admission (24 ± 7 *vs*. 21 ± 6 per min; p = 0.04), a higher heart rate (91 ± 20 *vs*. 85 ± 20 bpm; p = 0.04), a lower room air partial pressure of oxygen in arterial blood (73 ± 15 *vs*. 80 ± 17 mm Hg; p = 0.03), a higher cumulated volume of blood loss (360 ± 240 ml *vs*. 180 ± 150 ml; p < 0.0001), and a lower haemoglobin value (11.5 ± 2.6 *vs*. 13.3 ± 2; p < 0.001); they also had more often active bleeding on bronchoscopy (31/68 *vs*. 22/114, p = 0.0003), a first-line attempt at bronchial arteriography (66/73 *vs*. 81/123; p < 0.0001), a need for surgery (25/73 *vs*. 9/123; p < 0.0001) and specific aetiologies [mycetoma (11/73 *vs*. 3/123; p = 0.002) and cancer (22/73 *vs*. 11/123; p < 0.001)], but not a higher frequency of cardiovascular and pulmonary pre-existing diseases.

### Cause of haemoptysis

Bronchiectasis (n = 78, 40%), lung cancer (n = 33, 17%), active tuberculosis (n = 27, 14%) and mycetoma (n = 14, 7%) accounted for 87% of all causes. Emphysema (n = 10, 5%), pneumonia (n = 6, 3%), pulmonary embolism (n = 2, 1%) and miscellaneous causes (n = 5, 3%) accounted for the remaining probable causes. In 21 patients (11%), no cause was evidenced. The cause of bleeding was identified in 69% (n = 111/162) of patients at bedside when combining history, comorbid conditions, physical examination, chest-X-Ray and fiberoptic bronchoscopy findings, as compared with 91% (n = 148/162) after a further CT scan (p < 0.001). The CT scan examination was especially useful for diagnosing bronchiectasis.

### Management

All patients received conservative measures. Local (n = 6) or systemic (n = 37) terlipressin, mechanical ventilation (n = 3) and blood transfusion (n = 3) were administered to the most severe patients before referral. Forty-three (22%) patients were receiving aspirin, coumadin or clopidrogel that may have worsened the bleeding, and these drugs were temporarily stopped whenever possible. Broad-spectrum antibiotics were administered to 153 patients (78%). A fiberoptic bronchoscopy was performed within 24 hours of bleeding onset in most patients (n = 184, 94%). Diffuse and bilateral (n = 13) or localized endobronchial bleeding (n = 163) was evidenced in 176 patients (96%). The bronchoscopic findings revealed a localized active endobronchial bleeding in 53 patients and a localized endobronchial clotting in 41. Otherwise, a localized endobronchial bleeding was evidenced in the upper (n = 43) or lower bronchia (n = 26) without active bleeding or clotting. In the remaining 8 patients (4%), a few signs of endobronchial blood were present. Bronchoscopic techniques were combining blood aspiration and local instillation of cold saline lavage. Vasopressors were bronchoscopically delivered in 23 patients, and a balloon was placed in one patient.

**Figure 3:**
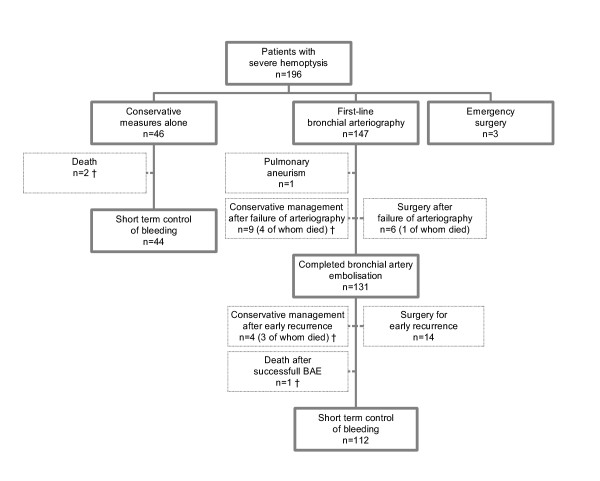
**Initial management and short-term outcome**. †Life sustaining therapy was withheld/withdrawn in 2/46 patients managed conservatively and in 10/147 patients in whom BAE was first attempted.

A first-line bronchial arteriography was attempted in 147 patients (75%), whereas 46 (23%) received conservative treatment. Emergency surgery was performed in 3 patients (bleeding of 700 ml revealing a cancer complicated by a cardiac arrest; bleeding of 300 ml revealing a cancer nearby the pulmonary artery; bleeding of 200 ml complicating repeated obstructive pneumonias in a patient diagnosed with a cancer) (Figure 3). The following parameters on admission were associated with the first attempt of arteriography as opposed to conservative treatment alone: a higher respiratory rate (23 ± 7 *vs*. 20 ± 4; p = 0.03), a greater amount of bleeding (290 ± 205 *vs*. 80 ± 50; p < 0.0001), a persistent bloody sputum (87/119 *vs*. 18/35; p = 0.02), an active bleeding on bronchoscopy (49/141 *vs*. 3/36; p = 0.002), the identification of a definite cause of haemoptysis (120/149 definite causes *vs*. 9/21 cryptogenic; p = 0.0005) and the absence of renal impairment (creatinin, μmol/l; 73 ± 22 *vs*. 82 ± 25; p = 0.03).

Technical failure of the attempted arteriography occurred in 15/147 (10%) patients, mostly those with mycetoma (n = 4) and cancer (n = 6). This led to either maintaining conservative measures in 9 patients or to surgery in six; 5 of these 15 patients died within the first month (4/9 patients managed conservatively and 1/6 undergoing surgery). In another patient, bleeding was related to a pulmonary artery aneurysm. Bronchial artery embolisation was eventually completed in 131/147 patients (89%) leading to an immediate control of bleeding in 106 patients (81%), 8 of whom had a secondary scheduled surgery (Figure 3).

Bleeding recurred in 7/46 patients (15%) managed conservatively, 2 of whom received BAE secondarily. Bleeding recurred in 35/131 patients (27%) receiving completed BAE. Haemoptysis recurred after 3 ± 3 days (range, 0 to 11 days) in 25 patients, who received conservative treatment (n = 4), BAE (n = 7) or surgery (n = 14). Mycetoma and cancer accounted for 50% of the early recurrences. There were 10 late recurrences (9 ± 4 months; range, 2 to 14 months) managed conservatively (n = 4) or with a second BAE (n = 3) or surgery (n = 3). Overall, surgery (pneumonectomy, n = 3; lobectomy, n = 11) was performed after 7 ± 7.5 days for early recurrences (mycetoma, n = 6; bronchiectasis, n = 3; pneumonia, n = 3; cancer, n = 2). A lobectomy was performed for late recurrences 12 ± 5 months after the initial episode.

Bronchial artery embolisation was associated with a 5% rate of complications (minor arterial dissection, n = 2; coronary ischemia, n = 2; chest pain, n = 1; transient neurological episode, n = 1; and dysphagia; n = 1). The outcome of these patients was uneventful without further intervention.

### Outcome

The lengths of ICU and hospital stay were respectively 5.4 ± 4.9 days (range, 0 to 47 days) and 10.7 ± 14 days (range, 0 to 142 days). Lengths of ICU (6 ± 5.4 days *vs*. 3.8 ± 2.5 days) and hospital stay (11 ± 14.4 days *vs*. 8.8 ± 12.4 days) were significantly longer for patients in whom a bronchial arteriography was first attempted, as compared with patients receiving conservative treatment alone (all p < 0.01). Therapy was withheld or withdrawn in 12 patients (6%), 7 of whom died in ICU. The ICU and hospital mortality rates were 4% (n = 8) and 8% (n = 15), respectively. At short term follow-up (one month), successful control of haemoptysis was obtained using completed BAE, conservative management or surgery in respectively 112 (57%), 44 (22%) and 22 (11%) patients. No recurrence of haemoptysis occurred in 116 (89%) of the 131 patients in whom BAE was completed, after a mean (median) follow-up duration of 20 (8) months.

## Discussion

Our study aimed at characterizing the clinical spectrum and the outcome of a large series of consecutive patients with severe haemoptysis requiring ICU admission in the early 2000's. The major aetiologies recorded were bronchiectasis, lung cancer, active tuberculosis and mycetoma. A simple set of clinical variables on admission combined with bronchoscopic findings were associated with attempting a first-line bronchial arteriography. This approach was applicable to 75% of our patients and led to an immediate control of bleeding in more than 80% of them. Although the median cumulated volume of haemoptysis averaged 200 ml on admission, the ICU mortality rate was low.

Haemoptysis accounted for up to 15% of our admissions. This high rate reflects in part the fact that both our unit and the department of radiology of our hospital are referral centres for haemoptysis. The major criterion for ICU admission is the amount of blood loss despite the lack of standardisation for quantifying it, since it is known to be related to death [5]. Respiratory failure, a substantial drop of haemoglobin level, and haemodynamic failure all obviously mandate ICU admission, although their occurrence is not specified in most studies. While the usual criteria of severity accounted for a relatively small subset of our patients, the median cumulated volume of haemoptysis averaged 200 ml on admission and chronic obstructive pulmonary disease and cardiovascular disease were frequent.

Bronchiectasis, active tuberculosis and idiopathic haemoptysis were the most frequent diagnoses among a French cohort of 56 patients with life-threatening haemoptysis recorded between 1986 and 1996 [6]. In a small recent series of 29 patients with massive haemoptysis requiring ICU admission in Singapore between 1997 and 2001, bronchiectasis, mycetoma, active tuberculosis and cancer were the main causes identified [9]. In our series, bronchiectasis (mainly secondary to inactive tuberculosis), cancer, active tuberculosis and mycetoma were the leading causes. Such a distribution of the causes of haemoptysis underlines the following points: 1) First, active tuberculosis still remains a common cause of severe haemoptysis in France; 2) Lung cancer appears to be an emerging cause of haemoptysis requiring ICU admission. The latter finding is at variance with previous studies by Mal et al. [6] and Ong et al. [9], which may be related to the smaller number of patients included in those previous studies [6,9], the study period [6] and the geographic location [9], as well as the current lesser restrictive policy for ICU admission of cancer patients.

The indications of emergency surgery have gradually been reduced, because of the reported 20–30% operative mortality rate and improvement in interventional radiology techniques [1-4,10]. Bronchial artery embolisation is now considered as the most effective non-surgical first-line treatment of severe haemoptysis [11,12], although there is no randomized trial in this field. Bronchoscopy-guided topical haemostatic tamponade therapy has also been demonstrated to control haemoptysis with varying success rates, using flexible or rigid bronchoscope for the instillation of procoagulant substances, local injection of adrenalin solutions, insertion of small calibre catheters or placement of oxidized regenerated cellulose [13].

In our series, a bronchial arteriography was first attempted in 75% of patients, whereas only 2% underwent emergency surgery. A relatively large subset of our patients (23%) was managed conservatively, based on our approach to assess the amount of blood loss and other criteria of severity on admission. Technical failure of arteriography has been reported in up to 20% of attempts, although a lower rate is expected with the use of micro catheters in the near future [6,9,14-17]. Moreover, bleeding recurrences after successful completed BAE range from 0% to 30% and may be influenced by the cause of haemoptysis [18-20]. In our series, the rate and causes of bronchial arteriography failure were similar. Haemoptysis recurred in 27% of patients. There were mostly early recurrences, two thirds of which were eventually controlled by surgery. According to an 'intent to treat analysis', a first-line arteriography was associated with an immediate control and a durable cessation of bleeding in 112 (57%) and 116 (59%) patients, respectively. Although other series reported higher immediate successful rates of BAE for controlling haemoptysis, ranging from 85% to 95% [16,18,20], it should be noted that no information was provided on patients in whom the procedure was not completed [21]. In our series, bleeding was controlled in 112/131 patients (85%) within the first month and in 116/131 patients (89%) after hospital discharge, when the procedure was completed.

Using a strategy including a routine assessment of the amount of bleeding with a standardized scale, and promoting BAE over surgery, the outcomes of patients were good. The ICU mortality rate was low, as reported in recent series of so-called life-threatening haemoptysis [6].

The limitations of our study are related to its retrospective nature and to the fact that it was conducted in a referral centre with an extensive experience of severe haemoptysis on a heterogeneous patients' group. Nevertheless, our study reports one of the largest series of medical inpatients over a short time period and may provide a useful framework for the therapeutic management of haemoptysis in this clinical setting.

To summarize, a multidisciplinary approach remains the cornerstone for the management of severe haemoptysis. Bedside clinical evaluation and early fiberoptic bronchoscopy may safely screen patients for initial BAE, including surgical candidates. In the latter, surgery should be postponed as much as possible during active bleeding and performed early after control of bleeding. Otherwise, surgery should be reserved to cases of failure of interventional radiology and/or uncontrolled bleeding despite embolisation. Further prospective studies are needed to confirm the safety and the reproducibility of such a therapeutic approach; this approach may also be influenced by the use of the multi detector row helical CT scan, which can depict accurately the bronchial and non bronchial arteries, prior to the embolisation.

## Competing interests

The author(s) declare that they have no competing interests.

## Financial support

None

## Authors' contributions

Dr Fartoukh had full access to the data and takes responsibility for the integrity of the data and the accuracy of the data analysis.

*Study concept and design*: Fartoukh, Cadranel.

*Acquisition of data*: Parrot, Louis, Fartoukh.

*Analysis and interpretation of data*: Fartoukh, Parrot, Mayaud, Cadranel.

*Drafting of the manuscript*: Fartoukh, Parrot, Carette, Khalil.

*Critical revision of the manuscript for important intellectual content*: Parrot, Khalil, Carette, Bazelly.

*Statistical analysis*: Fartoukh, Parrot, Cadranel.

*Study supervision*: Fartoukh.
